# Integrative modelling of pH-dependent enzyme activity and transcriptomic regulation of the acetone–butanol–ethanol fermentation of *Clostridium acetobutylicum* in continuous culture

**DOI:** 10.1111/1751-7915.12033

**Published:** 2013-01-21

**Authors:** Thomas Millat, Holger Janssen, Hubert Bahl, Ralf-Jörg Fischer, Olaf Wolkenhauer

**Affiliations:** 1 University of Rostock, Institute of Computer Science, Department of Systems Biology & BioinformaticsUlmenstr. 69, 18051, Rostock, Germany; 2 University of Rostock, Institute of Biological Sciences, Division of MicrobiologyA.-Einstein-Str. 3, 18051, Rostock, Germany; 3 Institute for Advanced Study (STIAS), Wallenberg Research Centre, Stellenbosch UniversityMarais Street, Stellenbosch, 7600, South Africa; † Department of Food Science and Human Nutrition, University of Illinois at Urbana-ChampaignUrbana, IL 61801, USA

## Abstract

In a continuous culture under phosphate limitation the metabolism of *Clostridium acetobutylicum* depends on the external pH level. By comparing seven steady-state conditions between pH 5.7 and pH 4.5 we show that the switch from acidogenesis to solventogenesis occurs between pH 5.3 and pH 5.0 with an intermediate state at pH 5.1. Here, an integrative study is presented investigating how a changing external pH level affects the clostridial acetone–butanol–ethanol (ABE) fermentation pathway. This is of particular interest as the biotechnological production of n-butanol as biofuel has recently returned into the focus of industrial applications. One prerequisite is the furthering of the knowledge of the factors determining the solvent production and their integrative regulations. We have mathematically analysed the influence of pH-dependent specific enzyme activities of branch points of the metabolism on the product formation. This kinetic regulation was compared with transcriptomic regulation regarding gene transcription and the proteomic profile. Furthermore, both regulatory mechanisms were combined yielding a detailed projection of their individual and joint effects on the product formation. The resulting model represents an important platform for future developments of industrial butanol production based on *C. acetobutylicum*.

## Introduction

*Clostridium acetobutylicum* is a prominent member of the physiologically heterogeneous group of strict anaerobic clostridia. Its acetone–butanol–ethanol (ABE) fermentation comprises the two characteristic metabolic states, acidogenesis and solventogenesis, which are characterized by their fermentation products. Interestingly, in a chemostat culture the metabolism of continuously growing cells is governed only by the external pH (Bahl *et al*., 1982; Fischer *et al*., 2006). Growing on sugars (e.g. glucose), during acidogenesis the predominant fermentation products are acetate and butyrate. This type of anaerobic metabolism is referred to as butyric acid formation, which enables the bacterium to gain the maximal amount of energy (up to 3.25 mole ATP per mole glucose) using substrate-level phosphorylation (Jones and Woods, 1986). The second metabolic state, solventogenesis, is dominated by the solvents acetone and butanol as fermentation products (generating up to 2 mole ATP per mole glucose). In continuous cultures under phosphate limitation ‘high’ pH values (above pH 5.2) induce acidogenesis, whereas ‘low’ pH values (below pH 5.1) give rise to solventogenesis. Ethanol, the third compound which is eponymous for the ABE fermentation, is produced in minor amounts in both phases, and is only slightly elevated during solventogenesis.

With respect to the environmental pH it is important to notice that *C. acetobutylicum* is unable to maintain a constant intracellular pH. Instead, the cells preserve a constant transmembrane pH gradient and, consequently, the intracellular pH of *C. acetobutylicum* follows the extracellular pH with the difference of ΔpH ≈ 1 (Gottwald and Gottschalk, 1985; Huang *et al*., 1985; Dürre, 2005). The changing intracellular conditions cause alterations of the cellular physiology (Grupe and Gottschalk, 1992). Several experiments using phosphate-limited continuous cultures have reported that the specific activities (Andersch *et al*., 1983), transcription rates (Grimmler *et al*., 2011) and concentrations (Janssen *et al*., 2010) of enzymes included in the clostridial ABE fermentation pathway depend on the external pH level. Coincidentally, acid-producing enzymes operate optimally at acidogenic pH levels (pH > 5.2), whereas solvent-producing enzymes operate optimally at solventogenic pH levels (pH < 5.1). Additionally, typical solvent-producing enzymes are synthesized only during solventogenesis, e.g. AdhE1 (Janssen *et al*., 2010). These experimental findings indicate that pH-dependent specific kinetic enzyme activity and pH-induced adaptation of the transcriptomic and proteomic profile regulates the metabolic state of *C. acetobutylicum*. However, the interplay of both contributions and their joint effect on the pH-induced metabolic shift are poorly understood.

Therefore, we focus on the impact of changing pH levels on the metabolic network of ABE fermentation considering experimental ‘omics’-data reported for continuous cultures, especially those published by Janssen and colleagues (2010) and Grimmler and colleagues (2011) which investigated changes on the transcriptomic and proteomic level between acidogenic (pH 5.7) and solventogenic (pH 4.5) steady states using bilaterally agreed standard operational procedures (SOP): see *Experimental procedures* for further details. In comparison with batch cultures, continuous cultures offer the important advantage of generating highly reproducible, reliable, and homogenous data as crucial prerequisite for global transcriptomic, proteomic and metabolomic studies. Furthermore, secondary growth and stress responses of cells growing in a batch culture might mask physiological differences (Hoskisson and Hobbs, 2005).

In the present manuscript, we concentrate on the influence of pH-dependent biochemical reactions on the product formation and the regulation of ABE fermentation in continuous cultures at steady state. Towards this end, theoretical formalisms, which have been reported for isolated enzymes (Michaelis, 1922; Alberty and Massey, 1954; Alberty, 2006), have been applied and metabolic branch points with pH-dependent specific enzyme activities and enzyme concentrations are investigated.

Our first step, to illustrate the importance of consistent information about the pH-dependent mechanisms, the impacts of the intracellular pH on kinetic and transcriptomic regulation are considered separately. Afterwards, we show that the combination of both regulatory mechanisms is responsible for the found antagonistic behaviour. Additionally, it is demonstrated how properties of pH-dependent profiles of specific activities, e.g. pH optimum and pH width, affect the steady-state growth of *C. acetobutylicum* in continuous chemostat cultures. Finally, we summarize and discuss our results, focusing on general and specific consequences for modelling microbial adaptation to environmental changes.

## Results

### The network of ABE fermentation

**Figure 1:**
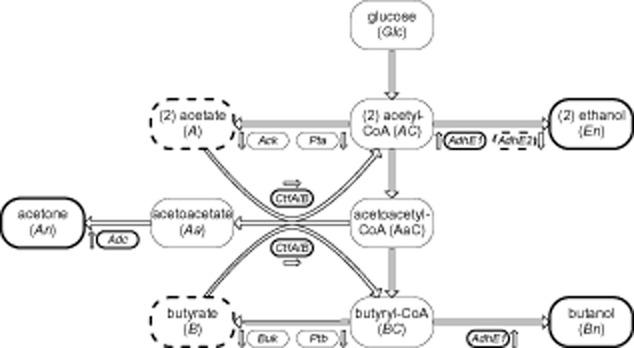
Simplified scheme of the metabolic network of ABE fermentation in *C. acetobutylicum*. During solventogenesis (low pH) the formation of solvents acetone and butanol and the concentrations of solvent-forming enzymes (solid frames) are increased, whereas the acids acetate and butyrate (dashed frames) are the dominating fermentation products during acidogenesis (high pH, see also Fig. 2). Ethanol (dotted frame) is fermented in similar amounts during both physiological phases by the antagonistically expressed pair of enzymes AdhE1/2. The abbreviations given below the arrows indicate the enzymes (see the main text for details). The arrows denote the relative pH-dependent changes of the specific activity from acidogenesis to solventogenesis, upward arrows symbolize an increase, downward arrows a decrease and horizontal arrows pH-independent activities.

A simplified scheme (Fig. 1) of the metabolic network of the ABE fermentation of *C. acetobutylicum,* relying on Jones and Woods (1986) and Lütke-Eversloh and Bahl (2011), is the basis for biotechnological processes focusing on biofuel and bio-solvent production (Papoutsakis, 2008; Lee *et al*., 2008b; Green, 2011; Jang *et al*., 2012). Although experimental evidence indicates a crucial role of the pH value, it is not understood in detail how the pH-dependent regulation is realized on biochemical (kinetic), transcriptomic and proteomic level. In the following, we introduce into the metabolic network with a focus on the relevant pH-dependent properties.

If the bacterium uses glucose as carbon and energy source this monosaccharide is transported into the cell via a phosphoenolpyruvate-dependent phosphotransferase (PTS) uptake system. Thereafter, glucose is metabolized via glycolysis (Tangney and Mitchell, 2007), which is expected to exhibit an insignificant pH dependence in the range from pH 7 to pH 4.5. Subsequently, the three key intermediates, acetyl-CoA, acetoacetyl-CoA and butyryl-CoA, are of particular interest for ABE fermentation with respect to different product formation during acidogenesis or solventogenesis. Thus, these intermediates are important branch points which direct the metabolic flow either to acid or to solvent formation.

The first key intermediate, acetyl-CoA, could be converted into acetate, ethanol, or condensed into acetoacetyl-CoA. Acetate is formed in two sequential reactions catalysed by phosphotransacetylase, Pta, and acetate kinase, Ack (Jones and Woods, 1986). The activity of the acetate kinase rapidly decreases to very low levels during solventogenesis (Andersch *et al*., 1983; Hartmanis *et al*., 1984), whereas no significant changes are detected on the protein level (Janssen *et al*., 2010). At the same time, ethanol is produced via an acetaldehyde and alcohol dehydrogenase activity. It is known that two NADH-dependent acetaldehyde/alcohol dehydrogenases, AdhE1 and AdhE2, play an essential role in the pH-induced metabolic shift (Dürre *et al*., 1995; Fontaine *et al*., 2002). This pair of enzymes is antagonistically expressed in a pH-dependent manner. Whereas the *adhE2* gene is transcribed during acidogenesis in continuous cultures and its gene product only promotes the formation of ethanol, transcription of the *adhE1* gene is induced in the solventogenic phase and its gene product seems to replace AdhE2 (Janssen *et al*., 2010; Grimmler *et al*., 2011). Interestingly, AdhE1 facilitates the formation of the two alcohols, ethanol and butanol.

The formation of the C3 and C4 fermentation products acetone, butyrate and butanol, respectively, starts from the second branch point, acetoacetyl-CoA. Formation of acetone is performed by the enzymes acetoacetyl-CoA transferase, Ctf, and acetoacetate decarboxylase, Adc (Jones and Woods, 1986; Dürre *et al*., 1995). Adc is generally known to be very pH-sensitive (Ho *et al*., 2009) and its specific activity increases 38-fold from acidogenesis to solventogenesis in *C. acetobutylicum* (Andersch *et al*., 1983). Additionally, active Adc is determined to be necessary for the uptake of acids via CoA-transferase after induction of solventogenesis (Hartmanis and Gatenbeck, 1984; Petersen and Bennett, 1990). However, in continuous cultures no significant differences of Adc protein concentrations could be observed at acidogenic and solventogenic steady-state culture conditions (Janssen *et al*., 2010). This suggests that the pH-dependent biochemical properties strongly affect the enzymatic activity of Adc (a pH-driven regulatory mechanism on the enzymatic kinetic level).

The last key intermediate, butyryl-CoA, initiates the formation of either butyrate or butanol. Butyrate is produced by sequential activities of phosphotransbutyrylase, Ptb, and butyrate kinase, Buk (Jones and Woods, 1986; Wiesenborn *et al*., 1989b). Both enzymes are most active during acidogenesis and their specific activities decline during solventogenesis, twofold for Ptb and sixfold for Buk (Andersch *et al*., 1983). This suggests that the internal pH is an important factor for their regulation (Bennett and Rudolph, 1995). Butanol is converted from butyryl-CoA in two steps by AdhE1. Importantly, experiments found that the *adhE1* gene is not expressed and no aldehyde/alcohol dehydrogenase activity was detectable in acid-producing cells. Its mRNA transcription rate and, consequently, protein concentration is highly increased during solventogenesis (Andersch *et al*., 1983; Fontaine *et al*., 2002; Janssen *et al*., 2010; Grimmler *et al*., 2011). These findings lead us to the conclusion that AdhE1 is regulated on transcriptional (transcriptome) and translational (proteome) level. With respect to butanol formation, it has to be mentioned that two further butanol dehydrogenases, BdhA and BdhB, have been identified in *C. acetobutylicum* that exhibit a strong pH-dependent activity with an *in vitro* optimum at acidogenic pH levels of pH 5.5 (Petersen *et al*., 1991). These isozymes are transcribed separately from each other and independently from other solvent-forming enzymes (Walter *et al*., 1992). However, recent experiments found that their transcriptional activity is low or even absent in comparison with AdhE1 in solventogenic continuous cultures (Janssen *et al*., 2010; Grimmler *et al*., 2011) suggesting that they contribute less to butanol formation under this experimental condition.

CoA-transferase, CtfA/B, has a fundamentally different role in clostridia compared with other bacteria (Wiesenborn *et al*., 1989a). It is induced during solventogenesis and responsible for the uptake of formerly excreted acids, their conversion to the respective CoA derivates (Andersch *et al*., 1983; Hartmanis and Gatenbeck, 1984), and, thus, partially essential for acetone formation. In contrast to other enzymes involved in acid or solvent formation, its specific activity seems to be insensitive to variations of the internal pH (Wiesenborn *et al*., 1989a).

In summary, the experimental findings document that the formation of acids and solvents is affected by pH-dependent specific enzyme activities, pH-dependent gene expression, and pH-dependent intracellular enzyme concentrations. Changes in the specific activity are associated with changes in the biochemical properties of the enzyme that could be merged in its kinetic coefficients, e.g. limiting rate and Michaelis–Menten constant. Hence, we refer to this regulatory mechanism as ‘kinetic regulation’. Naturally, the intracellular enzyme concentration affects the rate of the reaction. Changes in the concentration are usually assigned to changes in the expression of the encoding gene. Thus, we refer to this regulatory mechanism as ‘transcriptional regulation’. However, note that post-transcriptional processes may also modulate the intracellular amount of the enzyme.

**Figure 2:**
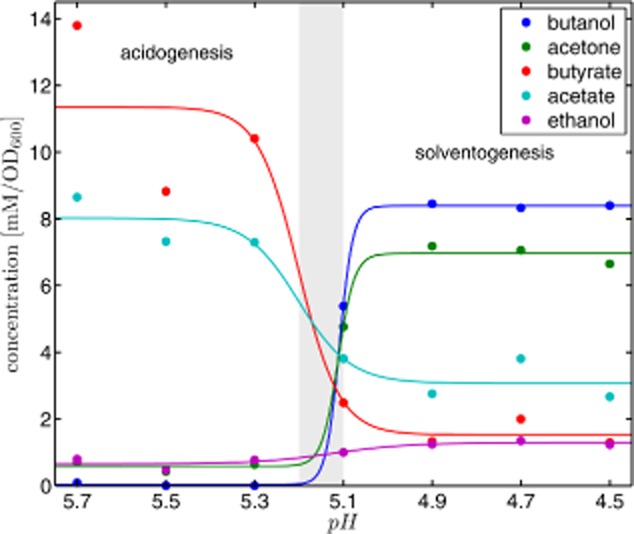
Fermentation products at steady state of continuously growing cells of *C. acetobutylicum* as a function of the external pH at seven different pH values between pH 4.5 and pH 5.7. Experimental data (dots) of product concentrations in mM are normalized to the optical density (OD_600_). From hyperbolic tangents (solid lines) fitted to the experimental data it follows that *C. acetobutylicum* exhibits acidogenic growth above an external pH of 5.2 and solventogenic growth below an external pH of 5.1. Between pH 5.1 and pH 5.2 an intermediate phase is observed which cannot be assigned to acidogenesis or solventogenesis. Data and parameters are summarized in Table 1, Appendix B, and Table 2, Appendix C respectively.

As a result of transcriptional and kinetic pH-dependent regulation, the product formation of *C. acetobutylicum* in continuous culture under phosphate limitation at steady state exhibits a switch-like behaviour as a function of the external pH as shown in Fig. 2. In this figure, the experimentally found pH-dependent product concentrations are compared with hyperbolic tangents fitted to the data. Using the inflection points of these functions, we have determined the critical pH levels that separate acidogenic and solventogenic behaviour, see Appendix C. We conclude that at a pH level above 5.2 *C. acetobutylicum* exhibits acidogenic growth, whereas below an external pH of 5.1 solventogenic growth is established. An intermediate phase is identified for the first time that cannot be associated to acidogenesis or solventogenesis. Furthermore, using the third derivative of the hyperbolic tangent, the width of the transition from acidogenesis to solventogenesis is found as 5.33 > pH > 5.07. Interestingly, the solvent concentration switches more sharply than the acids. These results refine the data of Bahl and colleagues (1982).

Only few kinetic models describing this fermentative process have been published. Papoutsakis (1984) developed a stoichiometric model and Desai and colleagues (1999) analysed the contribution of acid formation pathways to the metabolism of *C. acetobutylicum* ATCC 824 by using Metabolic Flux Analysis. In 2007, Shinto and colleagues presented a first kinetic simulation model to describe the dynamics of ABE fermentation in *Clostridium saccharoperbutylacetonicum* 1–4 in batch culture for glucose depletion. Haus and colleagues (2011) published a model of the pH-induced metabolic shift in *C. acetobutylicum* in phosphate-limited continuous culture considering an adaptation of gene expression and proteome composition to the changing external pH. Furthermore, several authors have investigated the overall flux through the whole solventogenic clostridial cell using genome-scale metabolic models (Lee *et al*., 2008a; Senger and Papoutsakis, 2008a,2008b; Milne *et al*., 2011; McAnulty *et al*., 2012). Among them, Senger and Papoutsakis (2008b) analysed the role of the proton flux, which depends on the intra- and extracellular pH level, on ABE fermentation in batch culture.

A major drawback of all existing published models is that they do not include the pH-dependent kinetic properties of the involved enzymes. The present work focuses on the consequences of pH-dependent regulations and its impact on steady-state ABE fermentation. Here, we illustrate the demand for complementary information about the interplay of pH-dependent regulation on the kinetic and the transcriptional level.

### pH-dependent enzyme kinetic reactions

**Figure 3:**
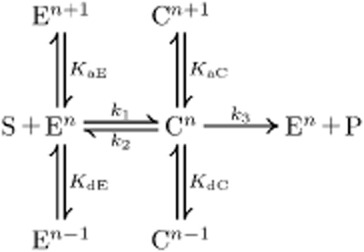
Reaction scheme for an enzyme kinetic reaction considering (de)protonation of the enzyme in its free form, as well as bound in the intermediary complex (Alberty and Massey, 1954). For the sake of simplicity, it is assumed that only the configuration with n bound hydrons facilitates the reaction. Thereby, the subscript ‘a’ denotes the association of a hydron and the subscript ‘d’ the dissociation. Both processes are determined by their dissociation constants *K*_a(E;C)_ and *K*_d(E;C)_. The number of bound hydrons is indicated by the superscript ‘*n*’.

Although it is known that the pH is one important parameter for enzymatic reactions, traditionally, pH-dependence is not reflected in the common Eq. 1 of the enzyme kinetic reaction:


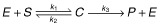
(1)

where the conversion of substrate *S* into product *P* is facilitated by enzyme *E*. During this conversion an intermediary complex *C* is formed (Segel, 1993; Bisswanger, 2002; Cornish-Bowden, 2004). The reaction becomes pH-dependent if it is considered that the association and dissociation of hydrons changes the structure of the enzyme and thus its specific activity. A schematic representation of such a pH-dependent enzyme kinetic reaction is shown in Fig. 3.

The incorporation of the pH-dependent association/dissociation of hydrons into the enzyme kinetic reaction results in a formal equivalent expression for the reaction rate (cf. Appendix A):


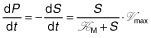
(2)

with a pH-dependent limiting rate 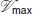 and a pH-dependent apparent Michaelis–Menten constant 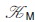. Now, both parameters are determined by the equilibrium of dissociation and association of hydrons. We assumed that the (de)protonation switches the enzymatic activity between an active (n bound hydrons) and an inactive state and are much faster than the enzymatic reaction, thus, these processes operate at their equilibrium states and can be described by their dissociation constants *K*_(a;d)E_ and *K*_(a;d)C_ respectively (Waley, 1953; Alberty and Massey, 1954; Dixon, 1973; Dixon *et al*., 1987) (Fig. 3). For more complex pH-dependent reaction schemes see, e.g. Segel (1993).

Applying the quasi-steady-state approximation to the enzyme–substrate complex and assuming that the total enzyme concentration is conserved, rate Eq. 2 is obtained. The resulting pH-dependent limiting rate is (Alberty and Massey, 1954; Dixon, 1973; Segel, 1993), see also Appendix A.


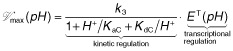
(3)

where *K*_aC_ and *K*_dC_ are the dissociation constants of the (de)protonation of the substrate-enzyme complex *H*^+^ is the hydron concentration. It is obvious from the equation above that the pH-dependent limiting rate fulfils the relation 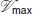 ≤ *V*_max_, where *V*_max_ = *k*_3_ *E*^T^ is the limiting rate of the standard Michaelis–Menten equation. Two multiplicative regulatory mechanisms contribute to this limiting rate: transcriptional regulation, adapting the enzyme concentration represented by the total enzyme concentration *E*^T^, and kinetic regulation represented by the pH-dependent rational expression.

**Figure A1:**
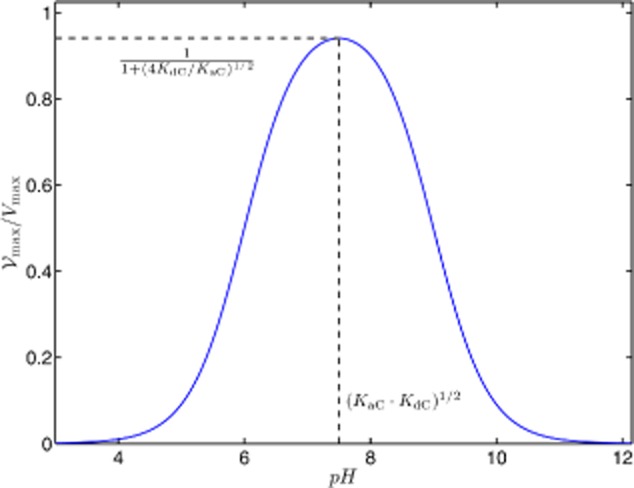
The apparent limiting rate *V* as a function of the pH value is bell-shaped. The height and the position of the pH maximum are determined by dissociation constants *K*_aC_ and *K*_dC_.

Due to the bell-shaped form of Eq. 3 (Alberty and Massey, 1954; Cornish-Bowden, 1976), we choose a simpler mathematical expression to describe the limiting rate as a function of the pH:


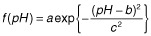
(4)

where *a* corresponds to the maximum, *b* to the location of the maximum and *c* to the half width of the curve (cf. Fig. A1, Appendix A). The pH-dependent Michaelis–Menten constant:


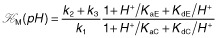
(5)

results from the multiplication of the standard Michaelis–Menten constant *K*_M_ = (*k*_2_ + *k*_3_)/*k*_1_ with a rational pH-dependent expression. Depending on the dissociation constants *K*_(a;d)E_ and K_(a;d)C_, this rational expression can be smaller than, greater than or equal to unity.

A further analysis of rate Eq. 2 shows that the pH-dependent limiting rate Eq. 3 has the greatest impact on the pH-dependent reaction rate. For the enzymes involved in ABE fermentation, systematic measurements of the pH-dependent limiting rate are unavailable except for Adc. However, due to the multiplicative contributions of kinetic and transcriptional regulation in Eq. 3, information about pH-dependent limiting rates are particular important to separate whether an enzyme is regulated on the level of activity (kinetic regulation) or on the level of transcription (transcriptional regulation) or both. Note, even in times of ‘omic'-analysis the sole measurement of gene expression provides an incomplete picture of the cellular dynamics in response to changing pH levels. For example, an increased protein synthesis could have two different effects (among others) on the limiting rate: First, the limiting rate is increased assuming a constant specific enzyme activity. Second, the limiting rate is preserved, because the raised protein synthesis compensates for a decreased specific activity.

The pH dependence of the apparent Michaelis–Menten constant Eq. 5 has to be considered for small substrate concentrations 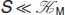. We here consider a pH-dependent limiting rate only. Assuming a pH-independent Michaelis–Menten constant, the first factor in rate Eq. 2 becomes a constant for fixed substrate concentration. As a consequence, the reaction rate Eq. 2 is directly proportional to the pH-dependent limiting rate and, therefore, shares the same functionality.

### Comparison of transcriptomic and kinetic regulation

**Figure 4:**
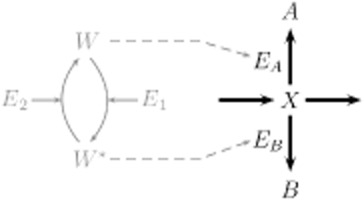
Scheme of the regulated metabolic branch point. The ‘branch’ metabolite *X* is either converted into the products *A* and *B* (e.g. acids and solvents) by the enzymes *E*_A_ and *E*_B_, respectively, or further intermediary metabolites. The specific activities and the concentrations of *E*_A_ and *E*_B_ depend on the pH level (see also *The network of ABE fermentation* in *Results*). Here, we assume that the enzyme concentrations are proportional to the amount of the pH-sensing protein W that changes its activation status, *W* and W*, in response to the pH level.

The product spectrum of ABE fermentation is determined by several branch points that direct the carbon flow either to acid or to solvent formation. Experimental evidence indicates that the product formation is regulated by changing enzyme levels and changing specific activities both affected by the pH level, illustrated in Fig. 4 (Andersch *et al*., 1983; Janssen *et al*., 2010). Thus it is determined by the pH-dependent rate Eq. 2. In our study, we assume that a pH-dependent sensory protein *W* regulates the expression of enzymes *E*_A;B_ facilitating the formation of products *A* and *B* from the branch point, e.g. butyrate and butanol (see also Fig. 4). Because we focus on the impact of changing enzyme concentration on the metabolic flux through the branch point, we assume further that their cellular amounts are directly proportional to state of protein *W*. However, several systematic comparisons of changes in gene expression and cellular protein concentration have revealed that both cellular levels are linked in a complex not necessarily linear manner (Maier *et al*., 2009). Thus, several exceptions to often assumed direct correlation between gene expression and proteome composition are described; see, e.g. Güell and colleagues (2009); Kühner and colleagues (2009) (*Mycoplasma pneumoniae*), Janssen and colleagues (2010) (*C. acetobutylicum*) and Güell and colleagues (2011) for a recent review of bacterial transcription.

In cells growing under steady-state conditions, the ratio of the active and inactive protein form is described by the Goldbeter–Koshland function (Goldbeter and Koshland Jr, 1981; Tyson *et al*., 2003):


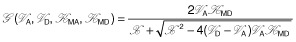
(6)

with


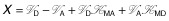


assuming negligible complex concentrations with respect to the protein concentrations in the derivation of this balance equation.

To characterize the effects of both regulatory mechanisms, we first investigate their isolated impact on the product formation assuming the limiting cases of pure transcriptional and pure kinetic regulation. For pure transcriptional regulation, the rational expression representing the kinetic regulation in Eq. 3 becomes independent on the pH level (approximately *k*_3_), so that Eq. 3 simplifies to an apparent standard limiting rate that is regulated by a pH-dependent total enzyme concentration only. Typically, strongly differing association and dissociations constants cause such a saturation-like behaviour. However, this approximation is restricted to a pH range. On the other hand, pure kinetic regulation assumes that only the first term in Eq. 3 depends on the pH level and that the enzyme concentration is constant, i.e. independent of the pH level. Such a behaviour was found for the protein abundance of acid-forming enzymes for which no significant changes of the concentration at steady state was found in continuous culture (Janssen *et al*., 2010), but remarkable variations of the specific activity were reported (Andersch *et al*., 1983). For the sake of demonstration using experimental information, we choose pH-optima *b*_B_ = 5.95 and *b*_A_ = 4.45 assuming that the acid- (B) and solvent-producing (A) enzymes optimally operate either during acidogenesis or during solventogenesis.

**Figure 5:**
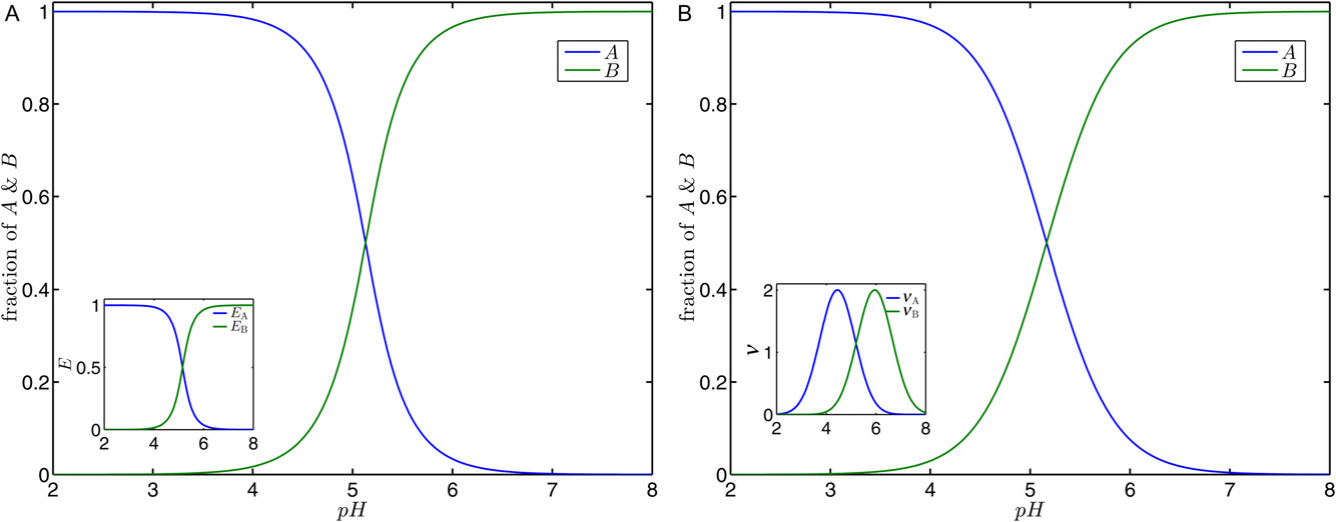
The fraction of products *A* and *B* with respect to the metabolite *X* as a function of the external pH value. We compare the effect of pure transcriptional (A) and pure kinetic regulation (B). In accordance with experimental results, we assume that the enzymes involved in activation/deactivation or product formation, respectively, operates optimally either in acidogenic or in solventogenic phase. The corresponding activities are shown in the insets as a function of the pH. The comparison of both regulatory mechanisms reveals that both could result in a similar switching behaviour which directs the product formation either to acids or to solvents in response to the external pH level.

In Fig. 5, we compare the fraction of product *A* and *B* produced from metabolite *X* per unit of time. This fraction is calculated as the ratio of the product formation and the overall conversion rate of *X*. Using product *A* as an example we obtain (an analogues expression can be derived for product *B*):


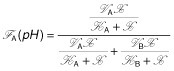


which can be transformed into


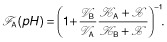
(7)

Two limiting cases emerge from the equation above. For 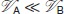 we find *F* ≈ 1 that implies that the product *A* is produced exclusively. On the other hand, for 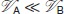 we obtain *F* ≈ 0, i.e. here *B* is solely formed as the product. As a consequence, if regulatory mechanisms exist that shift the system between both limiting situations, e.g. changing intracellular pH levels, a branch point exhibits switching behaviour. The level of product formation is adjusted by the Michaelis–Menten constants, although, their contribution is negligible for 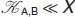, because then the second factor tends to unity. This confirms our previous statement that the pH-dependent limiting rates cause the switching behaviour.

What our analysis amounts to is that both regulatory mechanisms, pure transcriptional and pure kinetic regulation, independently result in a similar pH-dependent shift of the product spectrum. Remarkably, both mechanisms differ in their pH-dependent contribution to the limiting rate Eq. 3. Transcriptional regulation provides a sigmoidal behaviour reflecting the antagonistic induction of enzymes mediated by a pH-dependent sensory element (see inset Fig. 5A). A protein with two distinct states whose equilibrium distribution depends on the pH level, represented by a (de)activation cycle in Fig. 4, may act as such a sensor triggering changes in gene expression, see *Supporting information*, Figs S1 and S2. Using transcriptional regulation, the cell only increases the amount of an enzyme adapted to the current situation. In contrast, kinetic regulation is unrelated to modifications of the cellular composition. Here, the bell-shaped pH-depended limiting rates result in a changed metabolism (Fig. 5B). However, this requires that enzymes involved in those reactions are always present in the cell.

Furthermore, Eq. 7 states that the fraction and, thus, the steady state become pH-independent if both limiting rates behave identically as a function of the pH level.

In our example, the pH optimum of the enzyme *E*_A_ is at a smaller pH value than the activity of the enzyme*E*_B_. Hence, the product *A* is decreasing with increasing pH, whereas product *B* increases with the pH. Furthermore, the distance between the profiles is crucial for the shape of the pH-dependent steady-state response of the system. With increasing displacement between the optima, the pH-dependent steady states exhibit a transit from an adaptive to a highly non-linear switching behaviour, shown in *Supporting information*, Figs S1 and S3, where we varied the position of the pH optima.

### How the shape of the pH profile influences the metabolic shift

Aside from the position, the width of the pH profile influences the steady-state characteristics of the product spectrum. We therefore add the half width of the bell-shaped curve Eq. 4 as a further variable. The height of the curves remains constant. Furthermore, we introduce the displacement *d* to describe the distance between the maximum of the activities and the switching point *pH*_ref_ = 5.2. Then, the positions of the maxima are defined by:


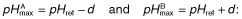
(8)

For positive displacements the activation is shifted to lower pH values and the deactivation to higher pH values. In case of negative displacements, the situation is mirrored.

**Figure 6:**
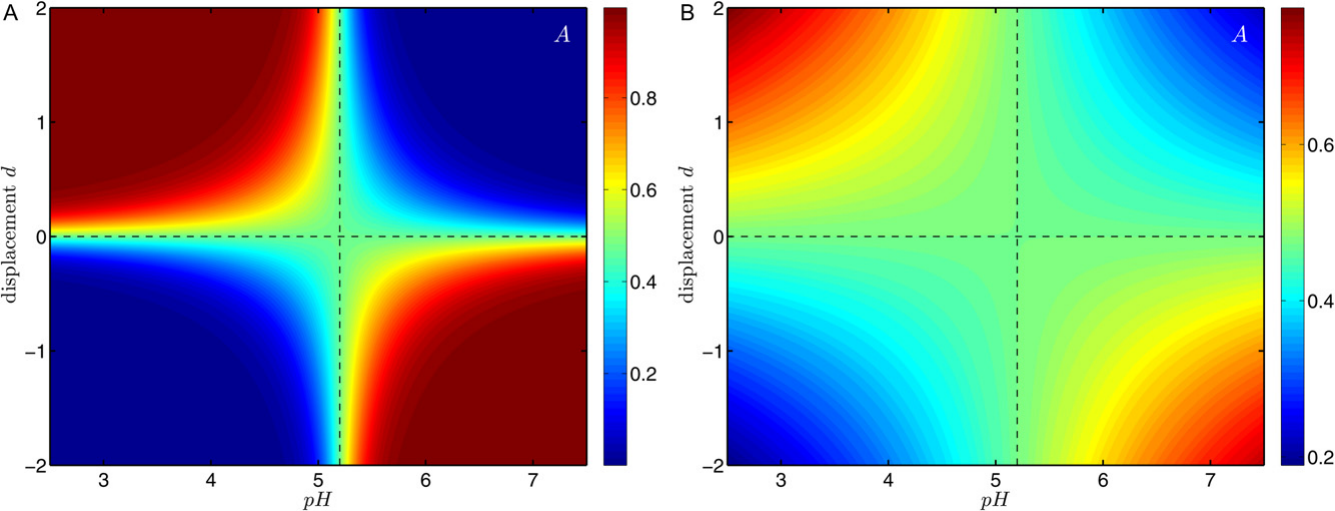
Contour plots of the ratio of product concentration *A* and the substrate concentration as a function of the external pH and the displacement *d*. Two situations are shown: (A) the pH-dependent limiting rates follow a Gaussian curve with a half width *c* = 1 and (B) *c* = 4. The switch-like shift from product *A* to product *B* is pronounced by smaller half widths.

To investigate the effect of the half width on the product spectrum, we use the limiting case of pure kinetic regulation as an example. We consider two different half widths, *c* = 1 and *c* = 4, and additionally change the shift between the two profiles. The contour plots for the concentrations of product *A* are plotted in Fig. 6. The corresponding contour plot for product *B* is given in *Supporting information*, Fig. S4. Furthermore, a similar analysis for the pure transcriptomic regulatory mechanism is shown in *Supporting information*, Fig. S2.

A comparison of both situations shown in the figure reveals that the width of the pH-dependent profile has a strong impact on the pH-dependent response of the branch point. Analogous to Fig. 5B, the system switches between both products if the pH is changed, but the transition phase (green area) is strongly affected by the width. For small half widths the product spectrum changes rapidly as from either formation of product *A* or *B*. Only at a small pH range, both products are produced simultaneously. Small half widths result in a highly non-linear pH-sensitive behaviour. Contrary, broad half widths reduce the sensitivity of the product spectrum to changing pH levels. Consequently, both metabolic products are formed over a wide pH range and their ratio varies less. Here, the broad half width compensates for different pH optima.

The position of the transition phase is determined by the switching point *pH*_ref_. Due to our simplifying assumption of two identical but shifted pH profiles, the transition is arranged symmetrically around this particular pH value. Note that profiles with different half widths break this symmetry, but would increase the number of free parameters.

The examples demonstrate that modelling of the pH-induced metabolic shift requires adequate information about the shape of the pH-dependent profile of transcriptional and kinetic regulation. In particular, separate experiments measuring transcriptomic and kinetic regulation allowing for a reasonable fit of the corresponding pH-dependent profile are needed to understand the complex relation between both regulatory mechanisms in the pH-induced transition from acidogenesis to solventogenesis. We note that transcriptomic data might be insufficient for that purpose, because they lack information about potential pH-dependent post-transcriptional regulations. Here, the combination of transcriptomic, proteomic and kinetic data obtained in standardized experimental set-ups could provide better insights into the metabolic phase transition.

### The joint effect of transcriptomic and kinetic regulation

Experimental evidence indicates that several solventogenic enzymes are induced during the shift from acidogenesis to solventogenesis in *C. acetobutylicum* (Fontaine *et al*., 2002; Janssen *et al*., 2010; Grimmler *et al*., 2011). Further biochemical studies revealed that their activities are strongly pH-dependent with a maximum during solventogenesis (Andersch *et al*., 1983; Ho *et al*., 2009). On the other hand, the specific activity of acid-forming enzymes rapidly decreases at solventogenic pH levels (Andersch *et al*., 1983; Hartmanis *et al*., 1984). These experimental results suggest that the pH-dependent induction of enzymes might be interpreted as a bifunctional replacement: On the one hand, the solventogenic enzymes re-establish the metabolic flow by circumventing the acid-forming enzymes. On the other hand, they prevent a further decrease of the pH level.

**Figure 7:**
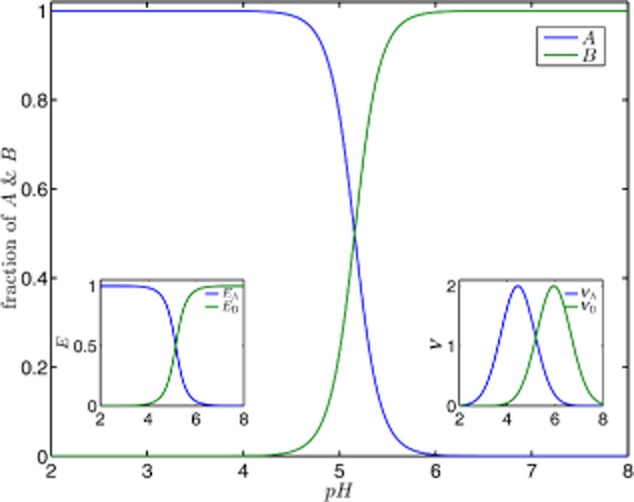
The ratio of product *A* and *B* as a function of the external pH for a metabolic branch point with regulated enzyme concentrations, see the schematic representation in Fig. 4. Here, we assume that enzyme *E*_A_ operates optimally at solventogenic (low) pH levels, whereas enzyme *E*_B_ is more adapted to acidogenic (high) pH levels. Thus, the respective enzymes are strongly induced either during solventogenesis or during acidogenesis to replace the enzyme that facilitating its associated reaction insufficiently during that phase. Cells are thus able to compensate for a dropped pH-depended kinetic activity and to re-establish a sufficient metabolic flow-through the ABE fermentation pathway. Furthermore, the product spectrum is then changed. The insets show enzyme abundances and pH-dependent limiting rates.

We now investigate the pH-dependent specific enzymatic activity and pH-dependent regulation of enzyme concentration, simultaneously. The steady-state ratio of the products *A* and *B* is shown in Fig. 7 as a function of the external pH level (see also *Supporting information*, Figs S5 and S6). Based upon the experimental observations, enzyme *E*_A_ shall be induced and shall be providing catalytic activity for low pH values (pH < 5.1), whereas the enzyme *E*_B_ is adapted to higher pH values (pH > 5.2). Such an antagonistically coupled pair of enzymes is able to restore the cellular metabolic function for changing pH levels. Because transcriptional and kinetic regulations exhibit non-linear characteristics as a function of the pH level, a combination of the two regulatory mechanisms might result in a further amplification of the pH-induced metabolic switch. Bearing in mind that *C. acetobutylicum* is unable to alter pH-dependent enzymatic properties (except on evolutionary timescales) transcriptional regulation provides the opportunity to modulate the cellular response to changing pH levels. Note that a homoeostatic configuration might also be established, where gene expression compensates for changes in kinetic efficiency. Thus, increased transcriptomic activity, followed by a rising intracellular enzyme concentration, is able to balance a pH-dependent decline of the catalytic efficiency. However, the non-linear characteristic of pH-dependent limiting rate Eq. 3 restricts homoeostatic regulation.

The factorial combination of transcriptomic and kinetic regulation in Eq. 3 introduces a further uncertainty to the interpretation of transcriptomic and proteomic data, because without reasonable information on the pH-dependent kinetics changing cellular compositions may be considered as a metabolic switch or a compensation for changing kinetic properties. Hence, a conclusion regarding the changes of the metabolic flow that finally determines the product concentration requires complementary information about the two regulatory mechanisms.

Our analysis has demonstrated that the joint contributions of transcriptomic and kinetic regulation provide a mechanistic explanation for the pH-induced metabolic shift in *C. acetobutylicum*. During the metabolic shift, the induced solvent-forming enzymes compensate for the kinetically caused reduction of metabolic flow-through the acid-forming pathways.

## Discussion

In response to changes in the environmental pH, the bacterium *C. acetobutylicum* is (reversibly) able to switch its metabolism between acidogenesis to solventogenesis in continuous cultures (Bahl *et al*., 1982; Fischer *et al*., 2006). During acidogenesis, the bacterium dominantly produces the acids acetate and butyrate, whereas the solvents acetone and butanol are the major products during solventogenesis. Small amounts of ethanol are formed during both phases. Several experiments have indicated that pH-dependent specific activities of acid- and solvent-forming enzymes contribute significantly to this phenomenon (Andersch *et al*., 1983; Hartmanis *et al*., 1984; Petersen and Bennett, 1990). Our own experimental studies, continuous culture experiments using phosphate limitation (Bahl *et al*., 1982; Fischer *et al*., 2006), proved that this metabolic adaptation involves changes not only of the enzyme activities, but also on the transcriptomic as well as on the proteomic, and metabolomic level (Janssen *et al*., 2010; 2012; Grimmler *et al*., 2011). However, the interplay and the individual contributions of each ‘omic’ level to the metabolic shift are only poorly understood. Thus, the motivation for this study was the question if these different regulatory domains are to be described mathematically on the basis of recent wet lab data. Furthermore, beyond existing models, in our work the pH was included as a distinct factor into the mathematical description. Undoubtedly, the pH as a single factor is able to control the product formation of *C. acetobutylicum* in phosphate-limited continuous cultures. Because *C. acetobutylicum* is unable to maintain a constant intracellular pH level (Jones and Woods, 1986; Dürre, 2005), changes of the external pH directly affect the intracellular pH (Gottwald and Gottschalk, 1985; Huang *et al*., 1985). Although some models of the metabolism of *C. acetobutylicum* have been developed, in none of them the influence of the pH level on kinetic activities was considered explicitly. Its effect on the proton flux through ABE network, investigated by Senger and Papoutsakis (2008b) for batch cultures, was neglected in this work.

Bearing in mind that the association and dissociation of hydrons to and from an enzyme catalysing a metabolic reaction in the ABE fermentation network results in a non-linear pH-dependent behaviour of the corresponding reaction rate, c.f. Segel (1993), we investigated the influence of changing pH levels on the product formation of branch points which direct the metabolic flow either to acid or solvent to formation. We demonstrated that two different regulatory mechanisms, transcriptional and kinetic regulation, influence the formation rate and that their impact on the limiting rate is of particular importance. Transcriptional regulation adapts the cellular enzyme concentration enabling the cell to modulate the metabolic flow by pH-dependent changes in gene expression. In contrast, kinetic regulation affects the properties of the enzyme directly and independently of the cell.

Most notably, our investigations, inspired by continuous culture experiments, clearly show that in principle pure kinetic and pure transcriptional regulations are able to explain the fermentation of products of *C. acetobutylicum* in response to changing pH levels (Fig. 2). This finding results from the fact that both mechanisms cause modifications in the ratio of the limiting rates (Eq. 7). This ratio mainly determines the fraction of product formation per unit time of a branch point. As a consequence, the estimation of the extent to which the two regulatory mechanisms control the branch point activity requires reproducible and reliable information collected using comparable experimental set-ups.

The analysis of the combined effect of kinetic and transcriptional regulation observed in the metabolic shift in *C. acetobutylicum* revealed that the pH-dependent transcriptional regulation adapts the protein composition. One logical response to changing pH levels is the replacement of inefficient enzymes, like AdhE2, by induction of additional enzymes suited to the new environmental condition. During solventogenesis, the specific activity of the acid-forming enzymes is low (Andersch *et al*., 1983), resulting in a reduced fermentation of acetate and butyrate. At the same time, the induced solvent-forming enzymes, optimally operating for solventogenic pH levels (Andersch *et al*., 1983), raise the rate of solvent formation. These findings further strengthen the importance of systematic experimental investigations of the limiting rate for dynamic modelling, including cellular protein abundance and rate coefficients. Note that the Michaelis–Menten constant, which has been the basis of several experiments in the past, contributes less to the dynamics of a biological system. In particular, the independent investigation of the pH-dependent concentration and pH-dependent specific activity of an enzyme could enhance the further improvement of our theoretical insight into the pH-induced metabolic switch in *C. acetobutylicum*, because the limiting rate is determined as the product of both pH-dependent functions, see also Eq. 7.

This leads us to the conclusion that the cellular response to changes of the extra- and intracellular pH relies on both kinetic and transcriptional regulation. Consequently, isolated and independent consideration of kinetic and transcriptional regulation may be misleading. Further experimental and theoretical studies therefore require reliable information about pH-dependent enzymatic properties and pH-induced changes on transcriptomic, proteomic and metabolomic levels. This information could provide the basis further model improvements and for a purposeful optimization of the bacterium for future industrial applications.

Furthermore, both regulatory mechanisms differ in their timescales. Kinetic regulation rapidly influences the enzymatic properties acting on a short timescale of (de)protonation, but the enzyme concentrations remains constant. Complementary to this, transcriptomic regulation involves several levels of cellular organization and is, therefore, slow in comparison with kinetic regulation. First, the state of a sensory protein is shifted in response to the change of the pH, e.g. by pH-induced conformational changes. This signal is mediated to the transcriptional machinery. Here, it triggers the induction or repression of genes which might result in a replacement of proteins like for the pair of aldehyde/alcohol dehydrogenases AdhE1/2. Thus, transcriptional regulation affects the reaction rate by adaptation of the protein concentration which is proportional to the timescale of protein synthesis and degradation respectively. This separation of timescales indicates an upper limit for the rate of environmental changes to which *C. acetobutylicum* is able to respond.

Although the present manuscript focused on changing pH levels and their impact on *C. acetobutylicum*, there are other environmental changes (e.g. osmotic and solvent stress) that are likely to affect kinetic properties, involving kinetic and transcriptomic regulation. In analogy to theoretical formalism used here, the consideration of limiting rates depending on environmental parameters like temperature and ion concentration into models of the microbial response to changing environmental conditions could improve our insights into the complex cellular adaptation which is more than just an alteration of the cellular transcriptomic profile. Thus, our results may apply to further phenomena as well.

## Experimental procedures

Recently, we developed a standard operating procedure (SOP) for the anaerobic growth of *C. acetobutylicum* ATCC 824 (COSMIC strain) at 37°C for the COSMIC consortium (http://www.sysmo.net) as documented by Janssen and colleagues (2010). In brief, pre-cultures were inoculated from spore stocks as previously described (Fischer *et al*., 2006) and the phosphate-limited chemostat experiments were performed with 0.5 mM KH_2_PO_4_ and 4% (w/v) glucose in the supplying medium using a BiostatB 1.5-l fermenter system (BBI, Melsungen, Germany) at 37°C (for further details see Fiedler *et al*., 2008). The dilution rate (respective generation time) was D = 0.075 h^−1^. Steady-state growth of *C. acetobutylicum* was performed for the following pH values: pH 5.7, pH 5.5, pH 5.3, pH 5.1, pH 4.9, pH 4.7 and pH 4.5. Therefore, the external pH was kept constant by automatic addition of 2 M KOH. Samples for measurement of fermentation products were taken when the respective cultures reached steady-state growth.

Optical density and fermentation products. The measurement of the optical density at 600 nm (OD_600_) and the analysis of the fermentation products (acetate, butyrate, butanol, acetone and ethanol) were accomplished as described elsewhere (Fischer *et al*., 2006).

## Appendix

### Appendix A: pH-dependent enzyme kinetics

For the sake of simplicity, we assume that only the enzyme with *n* bound hydrons may be enzymatically active (Alberty and Massey, 1954). The association and dissociation of a hydron deactivates the catalytic functionality of the enzyme. The corresponding pH-dependent reaction scheme is shown in Fig. 3. The enzyme concentration is restricted by the conservation law:

(A1)

where the superscript denotes the number of hydrons bound to the enzyme and the enzyme–substrate complex respectively. The total enzyme concentration *E*^T^ is assumed to be constant over the observation time. The binding of hydrons to the enzyme is much faster than the enzyme kinetic reaction. Hence, these reversible reactions are characterized by their corresponding dissociation constants *K_i_* :

After transformation with respect to enzymatic inactive forms and insertion into the conservation law Eq. A1, we obtain the expression:

(A2)

which depends on the enzymatic active forms and functions that describe the (de)protonation of enzyme and enzyme–substrate complex.

Applying the quasi-steady-state assumption for the enzyme–substrate complex, the enzyme kinetic reaction formally reduces to an apparent bimolecular reaction (Millat *et al*., 2007):

(A3)

with the Michaelis–Menten constant:

The definition of the Michaelis–Menten constant *K*_M_ as a ratio of the product of enzyme and substrate concentration and complex concentration allows a further reduction of Eq. A2. Transformation with respect to the complex concentration:

and insertion into Eq. A3 finally result in a pH-dependent expression for the reaction rate:

(A4)

with the pH-dependent limiting rate (Alberty and Massey, 1954; Segel, 1993):

and the pH-dependent apparent Michaelis–Menten constant:

Thereby, *V*_max_ = *k*_3_*E*^T^ is the common pH-independent limiting rate and *K*_M_ = (*k*_2_ + *k*_3_)/*k*_1_ the common pH-independent Michaelis–Menten constant. The maximum of the limiting rate is given by the relations:

## Appendix

### Appendix B: Product spectrum at steady state for different pH values

**Table A1:** Steady-state concentrations of the metabolic products of the ABE fermentation in *C. acetobutylicum*

	pH						
	Acidogenesis				Solventogenesis		
Products	5.7	5.5	5.3	5.1	4.9	4.7	4.5
Ethanol	3.9	2.4	4.0	4.7	6.1	7.0	5.9
Acetate	42.4	36.6	37.9	17.9	13.5	19.8	12.8
Butyrate	67.6	44.1	54.1	11.7	6.5	10.4	6.2
Acetone	3.4	2.1	3.3	22.4	35.2	36.7	31.9
Butanol	0.4	0.0	0.0	25.3	41.4	43.3	40.3
OD_600_	4.9	5.0	5.2	4.7	4.9	5.2	4.8

In a chemostat using phosphate limitation (Bahl *et al*., 1982; Fischer *et al*., 2006; Fiedler *et al*., 2008) only the pH value was varied. Product concentrations are given in mM. The concentrations strongly depend on the pH level, except for ethanol. The solvents acetone and butanol are mainly produced during solventogenesis (pH < 5.1), whereas the acids acetate and butyrate are produced during acidogenesis (pH > 5.2). Additionally, the optical density OD_600_ of the culture is given. It is applied to compensate for fluctuations in the clostridial population affecting the product concentrations.

## Appendix

### Appendix C: Product spectrum as a function of the pH level and determination of the pH-induced phase transition from acidogenesis to solventogenesis

We apply two distinct hyperbolic tangents to characterize the product spectrum of clostridial ABE fermentation in continuous cultures under phosphate limitation (Bahl *et al*., 1982; Fischer *et al*., 2006) as a function of the external pH level. The pH-dependent concentration of the solvents ethanol, acetone and butanol is described by:

In accordance with experimental data (Appendix B), this function represents a descending switch-like pH-dependent behaviour. The increase of the acids acetate and butyrate with increasing pH levels is described by the function:

The parameters of both functions are directly related to biological changes in ABE fermentation. Parameter *a* determines the change in the concentration between acidogenesis and solventogenesis. The minimal concentration either during acidogenesis or during solventogenesis is represented by *d*. The maximal concentration of a product, thus results from the sum *a* + *d*. The inflection point of these switch-like functions is determined by parameter *c*. We used it to define the pH-dependent metabolic phase of *C. acetobutylicum*. Finally, the parameter *b* is a measure for the steepness of the hyperbolic tangents. A transition range might be defined using the third derivative of the function. It defines the pH level where the product concentration is increased or decreased, respectively, by 2%. Then, the width of the transition range is proportional to the inverse of parameter *b*. The estimated parameters are summarized in Table2.

**Table A2:** The estimated parameters of the hyperbolic tangents used to fit the experimental data, Fig. 1

Product	*a*	*b*	*c*	*d*	*a* + *d*
Ethanol	0.636	11.7	5.12	0.653	1.29
Acetate	4.96	17.1	5.20	3.07	8.02
Butyrate	9.83	21.7	5.20	1.53	11.4
Acetone	6.38	54.9	5.11	0.583	6.96
Butanol	8.36	65.7	5.11	0.0272	8.39

The parameters *a* and *d* have the unit mM/OD, *c* of the pH and *b* of the inverse pH.

## Supporting information

**Fig. S1.** The fraction of the active and inactive form of protein W as a function of the external pH value. In panel a, we assume that activation and deactivation are pH-dependent, but follow an identical function (*b* = 5:2). Consequently, the steady state becomes independent on the pH level. The pH-dependent profiles differ slightly in panel b. This results in adaptive behaviour of the sensory protein W (*b_A_* = 5.15 and *b_B_* = 5.25) Finally, we investigate the effect that (de)activation is facilitated optimally either at acidogenesis or at solventogenesis (*b_A_* = 4.45 and *b_B_* = 5.95). Then, the pH-dependent sensory protein exhibits a switch-like behaviour which could trigger a physiological phase transition. Because we assumed that the enzyme concentrations follow the pH-dependent sensory protein, a purely transcriptionally regulated metabolic pathway provides the same switch-like behaviour. **Fig. S2.** Contour plot of the fractions of the active form *W** (upper row) and inactive form *W* as a function of the external pH value and the displacement *d*. The protein concentration is normalized against the total concentration of the protein W. In dark red areas the inactive form is exclusive, whereas dark blue areas correspond to a negligible concentration. Two half widths are compared: (panel a) *c* = 1. The protein W occurs over wide ranges either in its active (dark red) or in its inactive form (dark blue). Only within a small area both states exists simultaneously (light blue). (Panel b) *c* = 4. Here the pH-dependent activities are very broad. Consequently, the protein W exists in both states, while their ratio varies within the pH-*d* plane. **Fig. S3.** The fraction of products *A* and *B* with respect to the metabolite *X* as a function of the external pH value. Three different situations are compared: (panel a) both enzyme activities are pH-dependent but identical (*b* = 5.2), (panel b) the maxima of the activities differ slightly (*b_A_* = 5.15 and *b_B_* = 5.25), and (panel c) the maxima differ strongly from each other (*b_A_* = 4.45 and *b_B_* = 5.95). Because both enzyme activities are pH-dependent but identical, the steady states of the products are pH-independent. The corresponding activities, as a function of the pH, are shown in the insets. Differing pH-dependent profiles of the specific activity result in a pH-dependent product spectrum. Eventually, a changing pH shifts the product spectrum from metabolite *A* to metabolite *B* and vice versa. **Fig. S4.** Contour plot of the ratio of product concentration *B* and the substrate concentration as a function of the external pH and the displacement *d*. Two situations are shown: (panel a) the pH-dependent limiting rates follow a Gaussian curve with a half width *c* = 1 and (panel b) *c* = 4. The switch-like shift from product *A* to product *B* is pronounced by smaller half widths. **Fig. S5.** The fraction of products *A* and *B* as a function of the external pH for a metabolic branch point with regulated enzyme concentrations. Here, the identical pH-dependent profiles result in a pH-independent product spectrum. **Fig. S6.** Contour plot of the ratio of concentration of products *A* and *B*, respectively, and the substrate concentration as a function of the external pH and the displacement between the enzyme activities. Two situations are shown: (panel a) the pH-dependent limiting rates follows a Gaussian curve with a half width *b* = 1 and (panel b) *b* = 4. The switch-like shift from product *A* to product *B* is pronounced by smaller half widths.
