# Supplementary material for: Integrative modelling of pH-dependent enzyme activity and transcriptomic regulation of the acetone–butanol–ethanol fermentation of Clostridium acetobutylicum in continuous culture
Source: Microb Biotechnol. 2013 Jan 21;6(5):526–39. doi: 10.1111/1751-7915.12033 (PMC3918155; doi:10.1111/1751-7915.12033)
Supplement: Supplementary file 1 — Fig. S1. The fraction of the active and inactive form of protein W as a function of the external pH value. In panel a, we assume that activation and deactivation are pH-dependent, but follow an identical function (b = 5:2). Consequently, the steady state becomes independent on the pH level. The pH-dependent profiles differ slightly in panel b. This results in adaptive behaviour of the sensory protein W (bA = 5.15 and bB = 5.25) Finally, we investigate the effect that (de)activation is facilitated optimally either at acidogenesis or at solventogenesis (bA = 4.45 and bB = 5.95). Then, the pH-dependent sensory protein exhibits a switch-like behaviour which could trigger a physiological phase transition. Because we assumed that the enzyme concentrations follow the pH-dependent sensory protein, a purely transcriptionally regulated metabolic pathway provides the same switch-like behaviour. Fig. S2. Contour plot of the fractions of the active form W* (upper row) and inactive form W as a function of the external pH value and the displacement d. The protein concentration is normalized against the total concentration of the protein W. In dark red areas the inactive form is exclusive, whereas dark blue areas correspond to a negligible concentration. Two half widths are compared: (panel a) c = 1. The protein W occurs over wide ranges either in its active (dark red) or in its inactive form (dark blue). Only within a small area both states exists simultaneously (light blue). (Panel b) c = 4. Here the pH-dependent activities are very broad. Consequently, the protein W exists in both states, while their ratio varies within the pH-d plane. Fig. S3. The fraction of products A and B with respect to the metabolite X as a function of the external pH value. Three different situations are compared: (panel a) both enzyme activities are pH-dependent but identical (b = 5.2), (panel b) the maxima of the activities differ slightly (bA = 5.15 and bB = 5.25), and (panel c) the maxima differ stro [file mbt0006-0526-sd1.pdf]

SUPPLEMENTARY MATERIAL

Pure transcriptional regulation - The pH-dependent sensory protein

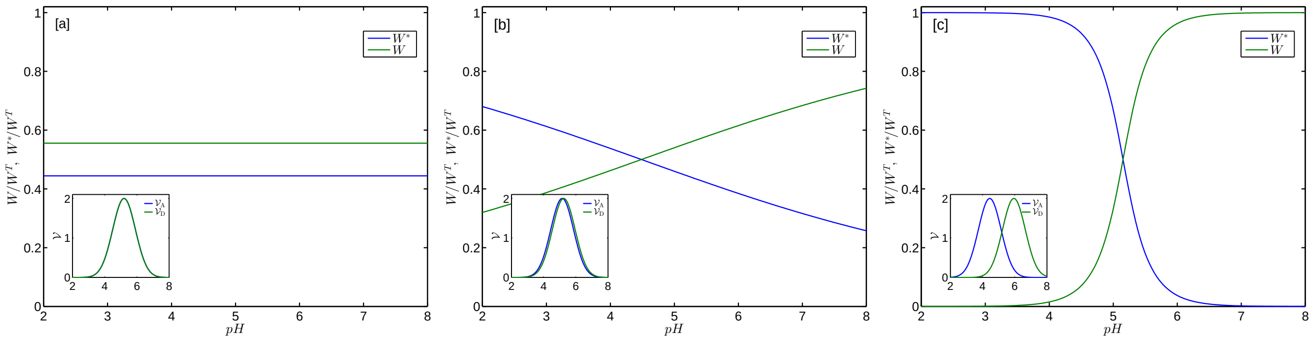

FIG. S1. The fraction of the active and inactive form of protein W as a function of the external pH value. In Figure [a], we assume that activation and deactivation are pH-dependent, but follow an identical function ($b = 5.2$). Consequently, the steady state becomes independent on the pH level. The pH-dependent profiles differ slightly in Figure [b]. This results in adaptive behaviour of the sensory protein W ($b_A = 5.15$ and $b_B = 5.25$) Finally, we investigate the effect that (de)activation is facilitated optimally either at acidogenesis or solventogenesis ($b_A = 4.45$ and $b_B = 5.95$). Then, the pH-dependent sensory protein exhibits a switch-like behaviour which could trigger a physiological phase transition. Because we assumed that the enzyme concentrations follow the pH-dependent sensory protein, a purely transcriptionally regulated metabolic pathway provides the same switch-like behaviour.

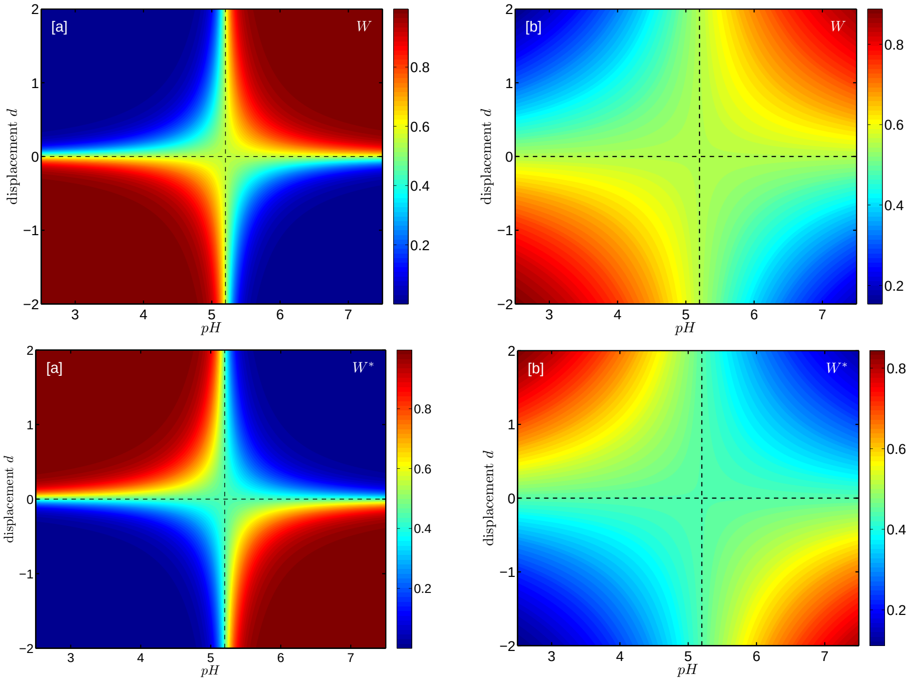

FIG. S2. Contour plot of the fractions of the active form W^* (upper row) and inactive form W as a function of the external pH value and the displacement d. The protein concentration is normalized against the total concentration of the protein W. In dark red areas the inactive form is exclusive, whereas dark blue areas correspond to a negligible concentration. Two half widths are compared: [a] $c = 1$. The protein W occurs over wide ranges either in its active (dark red) or in its inactive form (dark blue). Only within a small area both states exists simultaneously (light blue). [b] $c = 4$. Here the pH-dependent activities are very broad. Consequently, the protein W exists in both states, while their ratio varies within the pH-d plane.

Pure kinetic regulation - Branch point

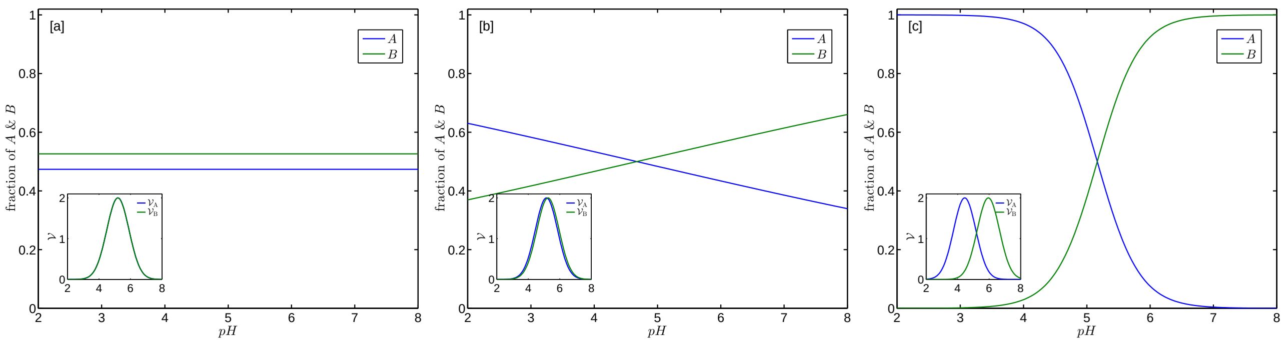

FIG. S3. The fraction of products A and B with respect to the metabolite X as a function of the external pH value. Three different situations are compared: [a] both enzyme activities are pH-dependent but identical ($b = 5.2$), [b] the maxima of the activities differ slightly ($b_{\mathrm{A}} = 5.15$ and $b_{\mathrm{B}} = 5.25$), and [c] the maxima differ strongly from each other ($b_{\mathrm{A}} = 4.45$ and $b_{\mathrm{B}} = 5.95$). Because both enzyme activities are pH-dependent but identical, the steady states of the products are pH-independent. The corresponding activities, as a function of the pH, are shown in the insets. Differing pH-dependent profiles of the specific activity result in a pH-dependent product spectrum. Eventually, a changing pH shifts the product spectrum from metabolite A to metabolite B and vice versa.

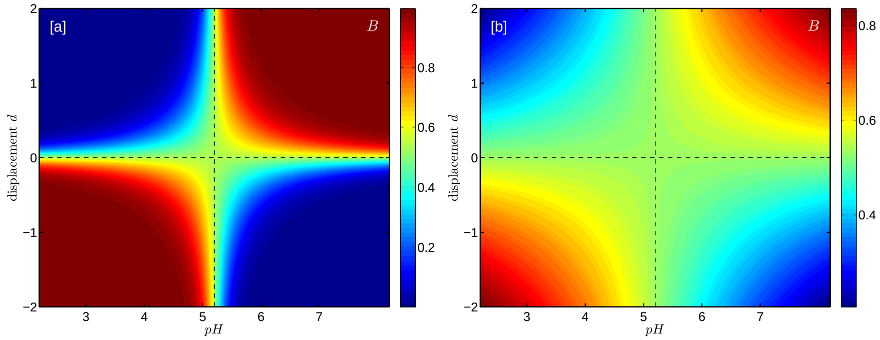

FIG. S4. Contour plot of the ratio of product concentration B and the substrate concentration as a function of the external pH and the displacement d. Two situations are shown: [a] the pH-dependent limiting rates follow a Gaussian curve with a half width $c = 1$ and [b] $c = 4$. The switch-like shift from product A to product B is pronounced by smaller half widths.

Transcriptional and kinetic regulation - Branch point

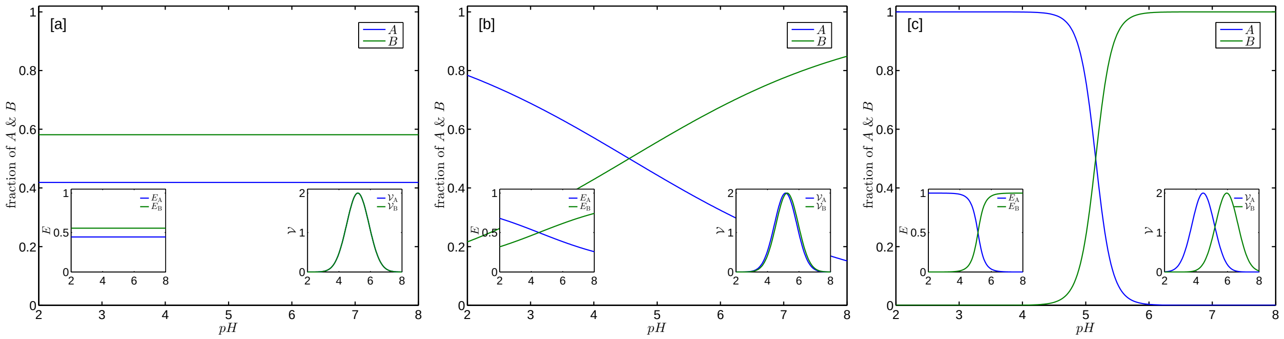

FIG. S5. The fraction of products A and B as a function of the external pH for a metabolic branch point with regulated enzyme concentrations. Here, the identical pH-dependent profiles result in a pH-independent product spectrum.

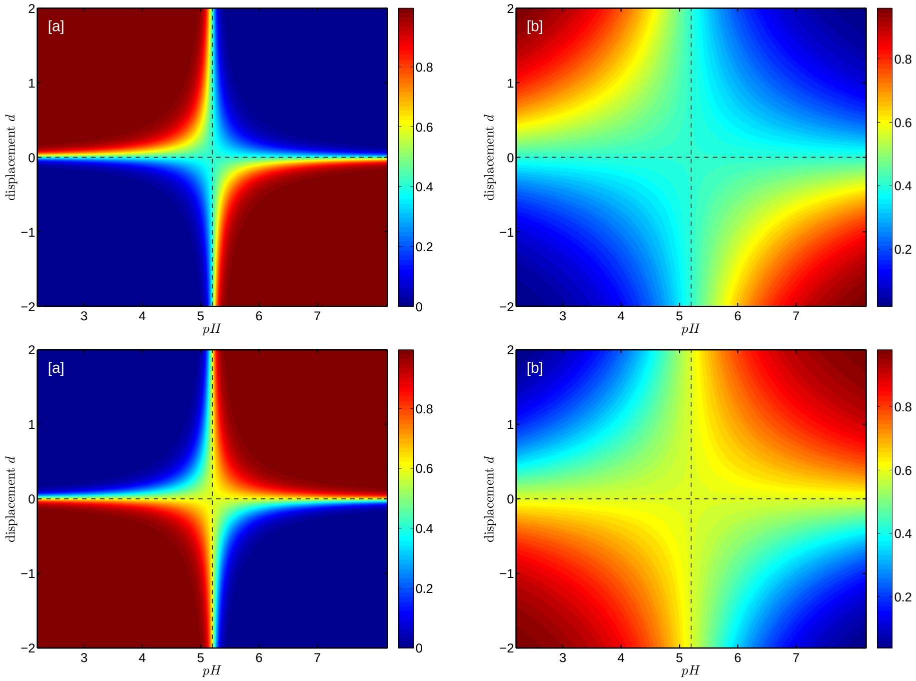

FIG. S6. Contour plot of the ratio of concentration of products A and B, respectively, and the substrate concentration as a function of the external pH and the displacement between the enzyme activities. Two situations are shown: [a] the pH-dependent limiting rates follows a Gaussian curve with a half width $b = 1$ and [b] $b = 4$. The switch-like shift from product A to product B is pronounced by smaller half widths.
